# Alternative methods to analyse the impact of HIV mutations on virological response to antiviral therapy

**DOI:** 10.1186/1471-2288-8-68

**Published:** 2008-10-22

**Authors:** Linda Wittkop, Daniel Commenges, Isabelle Pellegrin, Dominique Breilh, Didier Neau, Denis Lacoste, Jean-Luc Pellegrin, Geneviève Chêne, François Dabis, Rodolphe Thiébaut

**Affiliations:** 1 INSERM, Unit 897, Bordeaux, France; 2 University Victor Segalen, Bordeaux school of public health (ISPED), Bordeaux, France; 3 Bordeaux University Hospital, Department of Virology and EA 2968, University Victor Segalen, Bordeaux, France; 4 Bordeaux University Hospital, Department of Clinical Pharmacokinetics and Pharmacy and EA2968, University Victor Segalen, France; 5 Bordeaux University Hospital, Department of Internal Medicine and Infectious Diseases, Bordeaux, France

## Abstract

**Background:**

Principal component analysis (PCA) and partial least square (PLS) regression may be useful to summarize the HIV genotypic information. Without pre-selection each mutation presented in at least one patient is considered with a different weight. We compared these two strategies with the construction of a usual genotypic score.

**Methods:**

We used data from the ANRS-CO3 Aquitaine Cohort Zephir sub-study. We used a subset of 87 patients with a complete baseline genotype and plasma HIV-1 RNA available at baseline and at week 12. PCA and PLS components were determined with all mutations that had prevalences >0. For the genotypic score, mutations were selected in two steps: 1) p-value < 0.01 in univariable analysis and prevalences between 10% and 90% and 2) backwards selection procedure based on the Cochran-Armitage Test. The predictive performances were compared by means of the cross-validated area under the receiver operating curve (AUC).

**Results:**

Virological failure was observed in 46 (53%) patients at week 12. Principal components and PLS components showed a good performance for the prediction of virological response in HIV infected patients. The cross-validated AUCs for the PCA, PLS and genotypic score were 0.880, 0.868 and 0.863, respectively. The strength of the effect of each mutation could be considered through PCA and PLS components. In contrast, each selected mutation contributes with the same weight for the calculation of the genotypic score. Furthermore, PCA and PLS regression helped to describe mutation clusters (e.g. 10, 46, 90).

**Conclusion:**

In this dataset, PCA and PLS showed a good performance but their predictive ability was not clinically superior to that of the genotypic score.

## Background

The development of HIV resistance mutations is one of the major problems for optimizing treatment of HIV-infected patients. Therefore, resistance testing before starting highly active antiretroviral therapy (HAART) or before switching to a new antiretroviral component is widely recommended [1-4] and now routinely implemented in industrialised countries. Resistance is due to mutations in the viral genome, e.g. mutations in the reverse transcriptase (RT), protease or integrase genes that cause resistance to nucleoside RT inhibitors (NRTIs) and non-nucleoside RT Inhibitors (NNRTIs), protease inhibitors (PIs), or integrase inhibitors, respectively. Genotypic and phenotypic resistance testing are the two commonly used tests. The impact of genotypic mutations on virological response in patients treated with a particular drug regimen are based on *in vitro *informations or on the virological response reported in patients who switched to that particular regimen. Before the initiation of an optimized treatment, a genotype of the main (major) patients' virus populations (only virus species present at >20–30% are detected and therefore analysed) is assessed. Statistical analyses aim at finding the baseline genotypic mutations associated with virological response in order to predict whether a patient who will switch to a similar regimen is resistant or not. Noteworthy, data are mostly analysed for the main drug of a given regimen only, i.e. NNRTI and/or PI.

However, traditional statistical analyses of the association between genotypic mutations and virological response are hampered by i) the high number of potential mutations, ii) the correlations between mutations and iii) the low number of patients usually available for this type of study. Specifically, the analysis of the effect of high number of mutations measured in a limited number of patients may lead to over-fitting issues. Hence, inflated variances result in non-significant associations. In order to circumvent these problems and to simplify the interpretation, genotypic mutations are summarised in a so-called genotypic score. This score is the sum of observed resistance mutations at baseline for the given drug in a given patient. The mutations composing the score are selected by different strategies [5,6]. The drawbacks of this analysis are that a preselection of mutations is required and that every mutation has the same weighting. Alternative strategies such as principal component analysis (PCA) and partial least square (PLS) regression have been suggested for the sake of size reduction of correlated predictors [5,7-9] and may present advantages to improve the description of associations between mutations. The two techniques do not lead to a selection of mutations but to a different weighting of each mutation presented in the dataset. We aimed at comparing these two strategies with the usual construction of a genotypic score using data from an existing study evaluating the impact of protease mutations on the virological response in patients switching to a fosamprenavir/ritonavir-based HAART [10].

## Methods

### Data

The Zephir study was designed to investigate the impact of baseline protease genotypic mutations in HIV-1 infected PI-experienced patients on virological response. All patients had baseline HIV-1 RNA levels >1.7 log_10 _copies/mL and switched to a ritonavir-boosted fosamprenavir-based HAART [10]. Patients included were followed at the Bordeaux University hospital and at four other public hospitals in Aquitaine, south western France, all participating to the ANRS CO3 Aquitaine Cohort. We used a subset of 87 patients with a complete baseline genotype and plasma HIV-1 RNA available at baseline and at week 12. Virological failure was defined as a HIV-1 RNA ≥400 copies/mL and <1 log_10 _copies/mL decrease of HIV-1 RNA between baseline and week 12 (virological success: HIV-1 RNA <400 copies/mL or ≥1 log_10 _copies/mL reduction). A mutation was defined as a difference between the amino acid sequence of the studied virus and the wild type (HXB2) virus. In total, we created 69 dummy variables (69 mutations among the 99 possible protease mutations were encountered at least once).

### Statistical analysis

#### Construction of a genotypic score

The genotypic score was created in two steps. The first step considered mutations with prevalences ≥10% and ≤90% [5] to assess their association with virological failure. Mutations associated with a p-value ≥ 0.01 (univariable logistic regression) were selected. Second, the backwards procedure selected the combination with the strongest association with virological response [6]. These m selected mutations were used to calculate the first genotypic score for each patient. For instance, a first set contains the six mutations V32, I47, I50, V77, I84 and L90. The score is defined as S = I_V32 _+ I_I47 _+ I_I50 _+ I_V77 _+ I_I84 _+ I_L90 _(S varying from 0 to 6). During the backwards selection procedure every mutation was removed one by one and all combinations of (m-1) mutations were investigated. The Cochran-Armitage test for linear trends in proportions was used to compare the probability of virological failure in patients having none to (m-1) mutations [11]. The combination providing the lowest p-value was kept and the procedure was repeated with all combinations of (m-2) mutations. The procedure stopped when removal of a mutation did not result in a lower p-value.

We performed 200 bootstrap samples from the original data set to analyze the variability in mutations' selection. We assumed that variability in the selection of mutations due to the restricted sample size might essentially play a role in the first selection step. Therefore, a bootstrap analysis was performed only to the first selection criteria. In each sample the prevalence of each mutation was calculated. A univariable logistic regression was performed to determine the association of each mutation with virologic failure in each sample. Then we calculated the frequencies of selection of each mutation in the 200 bootstrap samples under the conditions mentioned above (prevalence between 10% and 90% and a p-value < 0.01 in univariable analysis).

#### Principal component analysis (PCA)

Each principal component is a linear combination of the original variables, with coefficients equal to the eigenvectors of the correlation or covariance matrix [7,9]. Principal components analysis determines components which are representing the variability of the mutations. The association between the principal components and the response variable was tested with the Wald test statistics of the estimated regression coefficient related to the principal components. We only tested principal components with an eigenvalue >2 reflecting that ≥3% of the variability of the mutations was explained. Any principal component was kept when it was related to the virological response using a logistic regression according to the Wald test.

#### Partial least square (PLS) regression

PLS regression is a technique widely used for dealing with numerous correlated explanatory variables [8,12]. PLS regression aims also at identifying components explaining as much as possible the variance of the predictor variables. These components are simultaneously correlated with the response variable. Over-fitting issues were controlled with a leave-one-out cross-validation during the construction process. The number of factors chosen is usually the one that minimizes the predicted residual sum of squares (PRESS) [13].

#### Comparison

The probability of virological failure at week 12 was studied using a logistic regression model adjusted for either the genotypic score or the principal components or the PLS components as explanatory variables. The performance of each strategy was compared using the cross-validated AUC [7,8]. We used 5-fold cross-validation. We split the dataset in five equal parts. That way we selected five times a dataset with 1/5 of the patients as 'validation set' and the remaining 4/5 of the patients served as 'test set'. In the test set, we determined i) the genotypic score ii) the principal components and iii) the PLS components. The selected mutations were then used to calculate the genotypic score for the patients included in the validation set. The weights for each mutation derived by PCA and PLS were applied to calculate the score of the principal component and the PLS component respectively for the patients of the validation set. For each validation set the AUC under the ROC curve was calculated by means of a logistic regression for the three different methods. Thus, we obtained for each method 5 AUCs and the cross-validated AUC was calculated as the mean of these 5 AUCs. This approach allows to avoid over-fitting because the performance of the methods is tested in a subset of patients that were not used to determine the genotypic score and the weights of mutations in the PCA and PLS components.

Statistical analyses were performed using SAS^® ^version 9.1 software (SAS Institute, Inc., Cary, NC). We used the procedures PROC PRINCOMP for principal component analysis and PROC PLS for partial least square regression. Principal components and PLS components were determined considering all mutations being present in at least one patient.

## Results

Study population characteristics have been reported before [10]. We used a subset of 87 patients with a complete baseline genotype and plasma HIV-1 RNA available at baseline and at week 12. Virological failure was observed in 46 (53%) patients at week 12. Mutations at codon 63 had the highest prevalence in this population 80% followed by mutations at codons 10 (58%), 71 (51%), 46 (47%), 54 (47%), 37 (47%), 35 (41%), 82 (40%) and 90 (40%). Mutations at codons 11, 12, 13, 14, 15, 19, 20, 32, 33, 34, 36, 41, 43, 47, 55, 57, 60, 61, 62, 64, 69, 72, 73, 77, 84, 89 and 93 had prevalences between 10% and 40%. Mutations at codons 10, 46, 54, 82 and 90 showed the highest association with virological failure in univariable analysis (p < 10^-5^). All patients with virological failure presented a mutation at codon 84.

### Genotypic score

Among mutations occurring in more than 10% and less than 90% of the patients, 27, 18 and 11 mutations were selected according to p-value thresholds of < 0.25, < 0.05 and < 0.01, respectively. The backward selection procedure using the Cochrane Armitage trend test was started with the 11 mutations (10, 33, 36, 46, 54, 62, 71, 73, 82, 84, 90) selected with the most restrictive criteria (p < 0.01) to avoid computational issues. The stability of this selection step was checked on 200 bootstrap samples. Seven (10:100%, 46: 100%, 54: 100%, 71: 95.5%, 82: 97%, 84: 100%, 90: 96%) of the 11 mutations were selected in over 90% of the samples. The other four mutations were selected between 50% and 90% (33: 88%, 36: 68%, 62: 50%, 73: 68.5%). Mutations not included in the IAS list [14] were in general not selected in the bootstrap samples (exceptions: 19: 36.5%, 37: 19% and 41: 19%). This additional bootstrap analysis confirmed that mutations known to be associated with virological failure were chosen for further steps. Mutations (also known as polymorphisms) that also occur occasionally in untreated patients, thus generally without any relation to antiretroviral treatment, were chosen in less than 3% of the bootstrap samples.

During the backward selection procedure the following six mutations 10, 36, 46, 62, 84, and 90 were selected for the calculation of a genotypic score. The genotypic score calculated with these six mutations was significantly associated with virological failure (OR = 4.1 for a difference of one mutation, CI_95% _[2.4; 7.0]; p < 10^-4^; cross-validated OR = 4.9).

### Principal component analysis

**Figure 1:**
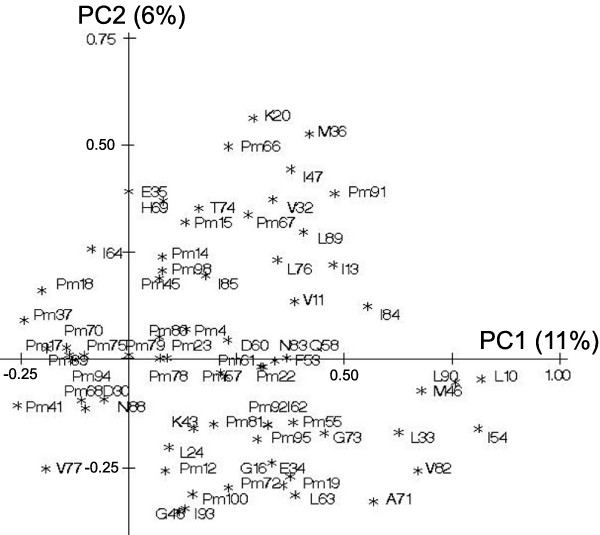
**Mutations on the first and second principal components. **All mutations having prevalences different from 0 are depicted. The wild type amino acid is cited before the codon of the mutation. Interpretation:  PC1: First principal component (representing 11% of the variability), PC2: Second principal component (representing 6% of the variability). Mutations are represented by the component when they are close to the corresponding axis. When two mutations are far from the center, then, if they are: i) Close to each other, they are significantly positively correlated; ii) If they are in a rectangular position, they are not correlated; iii) If they are on the opposite side of the center, then they are negatively correlated. When the mutations are close to the center, it means that some information is carried on other axes.

**Figure 2:**
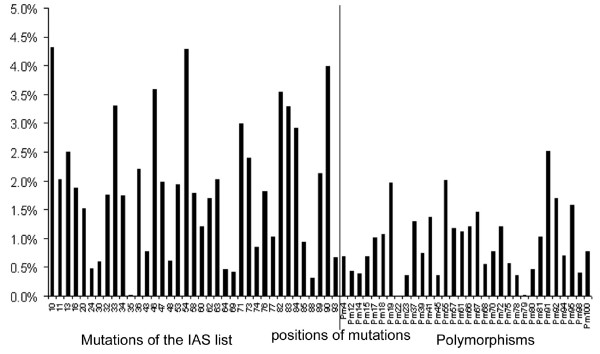
**Relative weights of each mutation to calculate the 'score' of the first principal component**. Black line: separation of mutations represented in the IAS list [14] and polymorphisms.

The first and second principal components explained 11% and 6% of mutations variability. Principal components accounted for a small variability overall. Therefore, their interpretation was difficult. The correlation of the mutations amongst them and to the principal components allowed identifying some clusters as for example mutations 10, 46 and 90 or mutations 32 and 47 already known to be associated together (figure 1). Figure 2 represents the relative weight of each mutation in the dataset to calculate the first principal component. The relative weight of each mutation to calculate the PCA 'score' ranged between 0% (e.g. mutation at codon 22) and 4.3% (e.g. mutations at codons 10 and 54). The sum of the relative weights of mutations represented in the IAS list was 70%, meaning that mutations of the IAS list contributed the most to calculate the first principal component. The mutations at the following six positions 10, 33, 46, 54, 82 and 90 contributed mostly to the first component (figure 2). Among others, mutations at positions 77, 88 and 30 contributed with a negative scoring coefficient to the first component, meaning that the presence of such mutation would decrease the value of the score. Medians of the first and the second principal component were -0.10 (IQR: -0.5–0.84) and 0 (IQR: -0.53–0.40), respectively. The first principal component was significantly associated with virological failure with an OR of 11.9 (CI_95% _[4.8; 29.7], p < 10^-4^) for a difference of one unit whereas the second was not OR = 1.1 (CI_95% _0.7; 1.7, p = 0.62).

### Partial least Square

**Figure 3:**
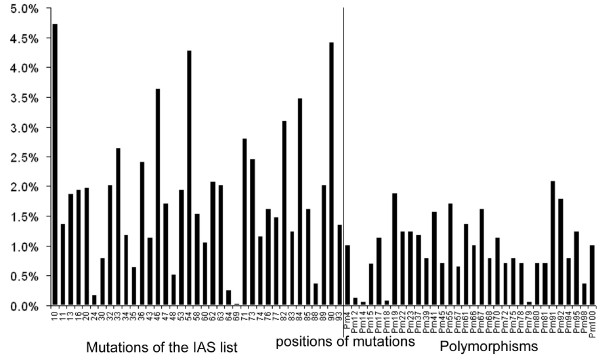
**Relative weights of each mutation to calculate the 'score' of the first PLS component**. Black line: separation of mutations represented in the IAS list [14] and polymorphisms.

One PLS component was chosen according to the PRESS criterion. This component explained 11% of the variability of the mutations and 60% of the variability of the response variable. The median of the first PLS component was -0.17 (IQR: -2.69–2.64). This PLS component was significantly associated with virological failure OR = 2.6 (CI_95%_1.8; 3.9 p < 10^-4^). Figure 3 represents the relative weight of each mutation in the dataset to calculate the first PLS component. Mutations at positions 10, 46, 54, 82, 84, and 90 had the highest contribution to the calculation of the first component (figure 3). Negative weight for the calculation of the first PLS component was amongst others given by mutations 77, 30 and 48. Mutation at codon 69 contributed with the smallest relative weight (0.03%) and mutation at codon 10 with the highest (4.7%). The contribution of mutations included into the IAS list was 69% (i.e. the sum of relative weights). Thus, mutations already known to be associated with virological failure were given more weight than polymorphisms (mutations that also occur occasionally generally without association to antiretroviral treatment).

### Comparison

**Figure 4:**
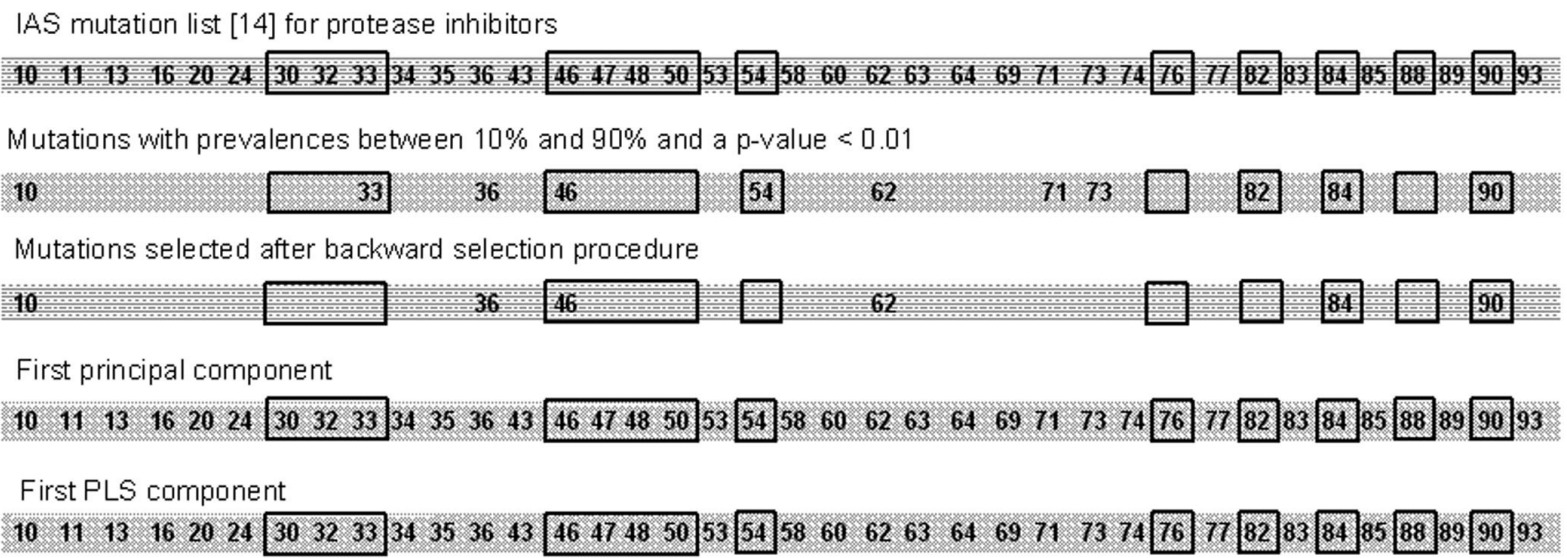
**Codons of mutations taken into consideration by the presented methods to predict virological failure(Codons at which polymorphisms occur are not depicted)**. The IAS mutation list shows all codons which have been described to be related with resistance to any of the protease inhibitors. Black boxes: Codons where major mutations occur.

We compared the results of the PCA and PLS with the results obtained using the classical strategy to build a genotypic score. Mutations 10, 46 and 90 were found among the six mutations contributing with the highest weight for the calculation of the first PC, the first PLS component and were selected for the genotypic score. Major mutations 54 and 82, which were found among the mutations with the highest association to virological failure in univariable analysis, were also found among the six mutations contributing with the highest weight for the calculation of the first PC and the first PLS component. In contrast, these two mutations were eliminated from the score during the backward selection procedure (figure 4). Therefore, one first advantage of methods based on PCA and PLS is that they helped in reducing the number of predictors without neglecting mutations that could play a significant role.

We compared the performance of these three methods with the area under the ROC curve. The cross-validated AUCs for the PCA, PLS and genotypic score were 0.880, 0.868 and 0.863, respectively. The model with the first principal component slightly outperformed the model with one PLS component. The predictive quality of the genotypic score was slightly lower than the two AUCs obtained for PCA and PLS but still showed a very good performance.

To compare the methods in an illustrative way we used a patient presenting the following 21 protease gene mutations at baseline: mutations at positions 33, 54, 82, 90 defined as major, mutations at positions 10, 13, 20, 35, 36 43, 53, 60, 63, 64, 74 defined as minor and mutations at positions 14, 15, 19, 37, 67, 98 defined as polymorphisms. Virological failure was observed for this patient. The genotypic score was S = I_10_+I_36_+I_90 _= 3 and the probability of virological failure was 77% using this score. The main difference between the genotypic score and the principal component value or the PLS component value is that with the latter methods we can take in consideration the fact that the patient has 21 protease gene mutations and give them different weights. For instance, the relative weights for mutations 10, 36, 90 were 4.4%, 2.2%, 4.1% and 4.7%, 2.4%, 4.4% for the PCA and PLS 'score', respectively (figure 2 and 3). The predicted probability of virological failure was 94% and 96% using the PC "score" and the PLS "score", respectively.

## Discussion

We investigated PCA and PLS regression to analyse associations between baseline protease mutations and virological failure. PCA and PLS are easily applicable because they are implemented in standard statistical analyses programs such as SAS (SAS Institute, Inc., Cary, NC).

We compared these two techniques with the construction of a genotypic score because they allow considering each mutation with a different weight. The objective of PCA is to find a set of new "latent variables" in form of a linear transformation of the original predictors. The properties of these latent variables are that they are uncorrelated and that they account for as much of the variance of the predictor variables as possible. PCA has been recently used to determine clusters of mutations in patients that were treated with at least one PI [15] and to predict the phenotypic fold change from genotypic information [16]. PLS regression reduces also a set of predictor variables to a set of uncorrelated "latent variables", the so-called PLS components. The main difference between the two techniques is that PLS also considers the strength of each mutation effect on the virological response to construct the components. Hence, these two methods can help solving the issues of the high number of predictors and their different effects. They may also help in describing the relationship between mutations by detecting potential groups of mutations. PLS was mentioned to be a useful analysing strategy for genotypic mutation data [5] but neither applications nor comparisons had been published yet.

In this study population, these two methods were able to identify some mutations that were expected to contribute with higher weights to virologic failure (e.g. mutations at codons 10, 82 and 90 which contribute to resistance to at least 7 of the 8 currently used PIs [5]). Furthermore, known clusters of mutations could be described. Recent papers including co-variation analysis [15,17-19] found some correlated pairs and clusters which are associated with a specific treatment. Two of them used PCA to visualise correlations of mutations. We identified some clusters of mutations, e.g. mutations at codons 10, 46, and 90 and at codons 33, 46, 54 and 82, which were also found to be correlated with each other. Mutations 32 and 47 had the highest correlation coefficient (r = 0.78) in this population and are known to be key mutations for amprenavir [20] and lopinavir [14]. The cluster of mutations at positions 10, 46, 90 [19] and a high correlation between 32 and 47 were also determined by Wu et al and Kagan et al [19,21]. The mutations 10, 33, 46, 54, 71, 82, 84 and 90 are separated from all other mutations by the PCA and are contributing with the highest weight to calculate this component. The cluster 10, 46, 54, 71, 90 was recently described [17] to appear under lopinavir treatment and these mutations are also related to amprenavir-resistance [22]. We found that PCA had indeed detected this latter cluster in our patient's population previously treated by lopinavir or amprenavir (25% and 32% of the patients, respectively). Furthermore, the fact that the principal component was related to virological response highlights that PCA can detect mutation clusters on the way to lopinavir and fosamprenavir resistance although principal component analysis did not consider the virologic response for the construction of the component. As mentioned above, PLS searches latent variables but takes into account the response variable. Consequently one might expect differences for the distribution of the weights given by the mutations. Actually, the mutations found to contribute the highest weight on the PLS component are almost the same. Among the six mutations contributing with the highest weight, mutations at codons 10, 46, 54 82 and 90 were found for the principal component and the PLS component. Mutation at codon 33 was found on the principal component, while mutation 84 was found on the PLS component. In addition, the mutations which contributed with a higher weight for the calculation of the first principal and first PLS components are those which showed the highest association with virological response in univariable analysis. In conclusion, the weightings of the mutations found were consistent across these alternative strategies. A possible explanation is that the patients were mainly pre-treated with two PIs known to induce similar mutation patterns than fosamprenavir. In other cases, PLS might outperform PCA when a drug induces completely different mutations since the virological response is considered during the construction of the component.

The above presented example (patient presenting 21 protease gene mutations) highlights the advantage of taking into account all mutations and giving them different weights by either PCA or PLS. This results in a better prediction of virological failure. After cross-validation the first principal component and the first PLS component only slightly outperformed the genotypic score in the prediction ability. However, it has to be stated that the cross-validated AUCs showed no clinical relevant difference. In this study population this might partly be explained by the fact that there was an explicit subset of mutations strongly associated with virological failure. This was also substantiated by the bootstrap analyses in which four of the six mutations remaining in the final genotypic score had been selected in over 95% of the bootstrap samples. This clear separation between mutations associated with virological failure from those which are not, could have facilitated the detection of a predictive subset using the classical strategy to construct a genotypic score.

One of the reasons to apply PCA and PLS analyses to these kind of data was that these approaches do not need a pre-selection of variables (i.e. mutations) as they are summarized in predictors. Hence, all mutations can be considered even when they are present in a small proportion of patients. Among others, the attempt to study these approaches was to study whether considering all mutations has an advantage and if mutations known to be associated with virologic failure are given higher weights. However, the slightly better performance of the alternative approaches may be simply linked with the use of a larger amount of information. This was the minimum expected gain of these approaches compared to the usual one.

Therefore, it would be very helpful to study the performance of PCA and PLS in other, potentially bigger, trials considering other antiretroviral regimen/patients.

## Conclusion

PCA and PLS regression were helpful in describing the association between mutations and to detect mutation clusters. PCA and PLS showed a good performance but their predictive ability was not clinically superior to that of the genotypic score.

## Appendix

### Aquitaine Cohort composition

**Scientific Committee**: J. Beylot, M. Dupon, M. Longy-Boursier, J.L. Pellegrin, J.M. Ragnaud and R. Salamon (Chair).

**Scientific Coordination**: M. Bruyand, G. Chêne, F. Dabis (Coordinator), S. Lawson-Ayayi, C. Lewden, R. Thiébaut.

**Medical Coordination**: N. Bernard, M. Dupon, D. Lacoste, D. Malvy JF. Moreau, P. Mercié, P. Morlat, D. Neau, JL. Pellegrin, and JM. Ragnaud.

**Data Management and Statistical Analysis**: E. Balestre, L. Dequae-Merchadou, V. Lavignolle-Aurillac.

**Technical Team**: MJ. Blaizeau, M. Decoin, S. Delveaux, D. Dutoit, C. Hanappier, L. Houinou, S. Labarrère, G. Palmer, D. Touchard, and B. Uwamaliya.

**Participating Hospital Departments (participating physicians)**: Bordeaux University Hospitals: J. Beylot (N. Bernard, M. Bonarek, F. Bonnet, D. Lacoste, P. Morlat, and R. Vatan), P. Couzigou, H. Fleury (ME. Lafon, B. Masquelier, and I. Pellegrin), M. Dupon (H. Dutronc, F. Bocquentin, and S. Lafarie), J. L. Pellegrin (O. Caubet, E. Lazaro C. Nouts, and J. F. Viallard), M. Longy-Boursier (D. Malvy, P. Mercié, T. Pistonne and C. Receveur), J. F. Moreau (P. Blanco), J. M. Ragnaud (C. Cazorla, D. Chambon, C. De La Taille, D. Neau, and A. Ochoa); Dax Hospital: P. Loste (L. Caunègre); Bayonne Hospital: F. Bonnal (S. Farbos, and M. C. Gemain); Libourne Hospital: J. Ceccaldi (S. Tchamgoué); Mont-de-Marsan Hospital: S. de Witte.

## Abbreviations

ANRS: Agence Nationale de Recherche sur le SIDA; AUC: Area under the receiver operating characteristics curve; CI: Confidence interval; HAART: Highly active antiretroviral therapy; HIV: Human immunodeficiency virus; IAS: International AIDS society; IQR: Interquartiles range; NNRTI: Non-nucleoside reverse transcriptase inhibitor; NRTI: Nucleoside reverse transcriptase inhibitor; OR: Odds ratio; PC: Principal component; PCA: Principal component analysis; PLS: Partial least square; PRESS: Predicted residual sum of squares; RT: Reverse transcriptase.

## Competing interests

The authors declare that they have no competing interests.

## Authors' contributions

LW carried out the statistical analysis and drafted the manuscript. RT and DC participated in the statistical analysis and helped to draft the manuscript. IP, DB, DN, DL, JLP, GC and FD performed the clinical trial and helped to draft the manuscript. All authors read and approved the final manuscript.

## Pre-publication history

The pre-publication history for this paper can be accessed here:


